# Review of robotic systems for thoracoabdominal puncture interventional surgery

**DOI:** 10.1063/5.0180494

**Published:** 2024-04-01

**Authors:** Cheng Wang, Li Guo, Jianjun Zhu, Lifeng Zhu, Chichi Li, Haidong Zhu, Aiguo Song, Ligong Lu, Gao-Jun Teng, Nassir Navab, Zhongliang Jiang

**Affiliations:** 1 Hanglok-Tech Co. Ltd., Hengqin 519000, People's Republic of China; 2 Center of Interventional Radiology and Vascular Surgery, Department of Radiology, Zhongda Hospital, Medical School, Southeast University, Nanjing 210009, People's Republic of China; 3 State Key Laboratory of Digital Medical Engineering, Jiangsu Key Lab of Remote Measurement and Control, School of Instrument Science and Engineering, Southeast University, Nanjing 210096, People's Republic of China; 4 School of Computer Science and Engineering, Macau University of Science and Technology, Macau, 999078, People's Republic of China; 5 Guangzhou First People's Hospital, Guangzhou 510180, People's Republic of China; 6 Zhuhai People's Hospital, Zhuhai Hospital Affiliated with Jinan University, Zhuhai 519000, People's Republic of China; 7 Computer Aided Medical Procedures, Technical University of Munich, Munich 80333, Germany; 8 Computer Aided Medical Procedures, Johns Hopkins University, Baltimore, Maryland 21218, USA

## Abstract

Cancer, with high morbidity and high mortality, is one of the major burdens threatening human health globally. Intervention procedures via percutaneous puncture have been widely used by physicians due to its minimally invasive surgical approach. However, traditional manual puncture intervention depends on personal experience and faces challenges in terms of precisely puncture, learning-curve, safety and efficacy. The development of puncture interventional surgery robotic (PISR) systems could alleviate the aforementioned problems to a certain extent. This paper attempts to review the current status and prospective of PISR systems for thoracic and abdominal application. In this review, the key technologies related to the robotics, including spatial registration, positioning navigation, puncture guidance feedback, respiratory motion compensation, and motion control, are discussed in detail.

## I. INTRODUCTION

Cancers are a major contributor to disease burden worldwide, and projections forecast that global cancer burden will continue growing in the near decades.^1^ Routine screening and early diagnosis as well as early treatment have become an effective way to prevent cancer, greatly reducing the death risk of patients with malignant tumors.^2^ Currently, puncture intervention surgery plays an important role in tumor treatment and the main operating treatments include puncture ablation, particle implantation, and biopsy sampling.^3–5^ In the traditional puncture interventional surgery, clinicians obtain the location of patient's lesion according to the preoperative medical images and determine the entry point of the needle and the patient's bed position. During the operation, the clinicians need to perform several CT scans to confirm the insertion depth and the current insertion needle position.^6^ Then, the needle is adjusted by comparing the target position and the current needle tip's location, which is repeated until the target is reached.

**FIG. 1.**
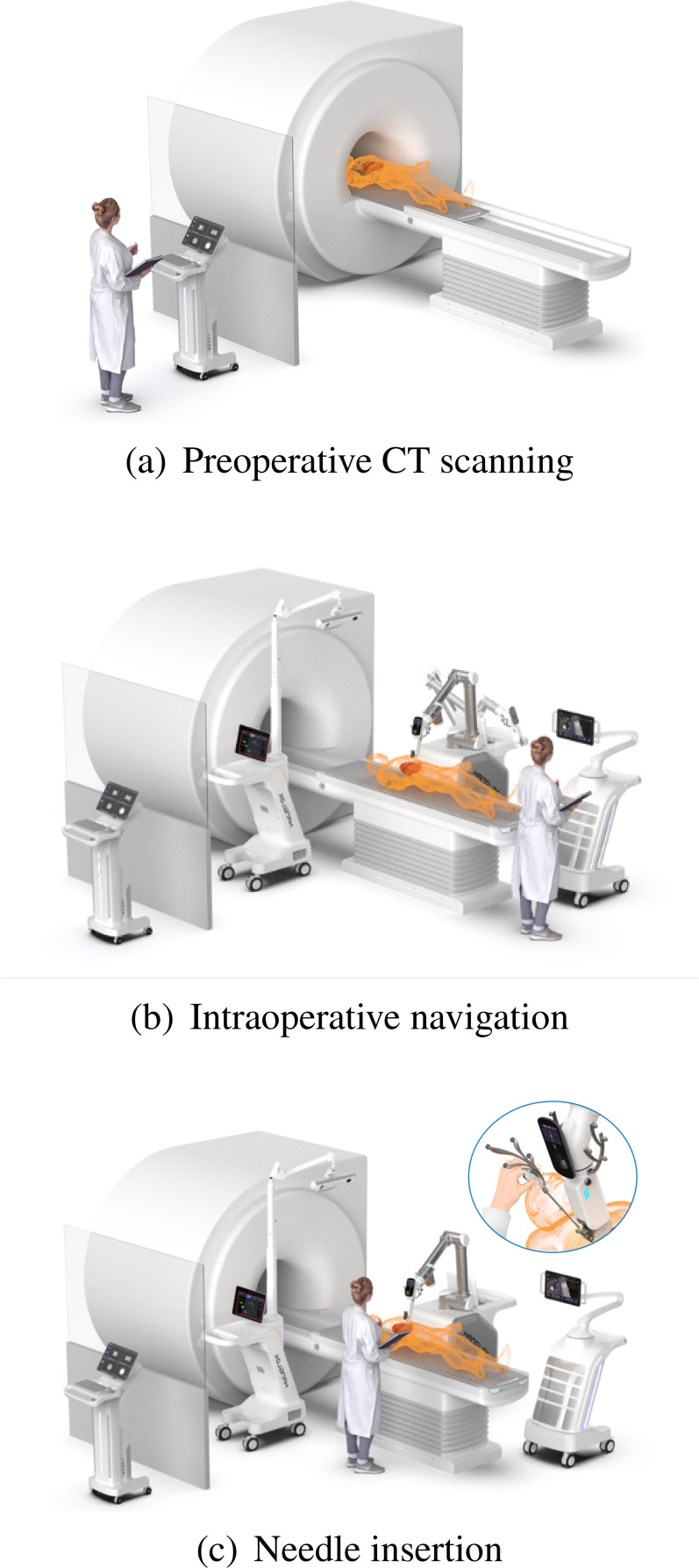
General procedures of robot-assisted puncture interventional surgery, including (a) preoperative CT scanning, (b) intraoperative navigation, and (c) needle insertion, reprinted with permission from https://www.hanglok-tech.cn/ (last accessed January, 2024). Copyright 2024 Hanglok-Tech Co. Ltd.

In recent decades, puncture interventional surgical robot (PISR) systems, primarily performing puncture procedures, have shown great potential in precise positioning, visualization imaging, and automatic control.^7^ In general, PISR systems consist of planning information generating module, navigation control module, sensing module, execution module, and central scheduling module. The operation of PISR systems generally includes three stages: modeling, planning, and execution. In the modeling stage, CT, MRI, and other medical image information of the lesion is collected by medical imaging equipment. Then, the 3D reconstruction is performed and presented to clinicians for identifying the lesion targets more accurately. In the planning stage, the spatial systems of robot, image, and patient are coordinated and registered before the path is planned. In the execution stage, the manipulator moves to the recommended entry point according to the planned path. The whole process is shown in Figs. 1(a)–1(c). However, due to the lack of real-time positioning of the needle tip, the interventional clinicians need at least twice CT scans to adjust the position of needle to reach the target location and the insertion process of needle relies on the clinicians' experience. Based on the application scenario, PISR systems can be categorized into orthopedic robotic surgery systems and solid tumor robotic surgery systems. This means that the patient's respiratory motion and natural movements intraoperatively hugely affect the positioning accuracy and puncture effect,^8^ especially for solid tumor robotic surgery. Additionally, the advancement of needle in flexible tissues, such as liver and stomach, is subjected to uneven friction force and cutting force, causing positioning accuracy errors.^9^ These issues hinder the further development of PISR and should be tackled with intelligent and reliable techniques.

Generally, the primary tasks of PISR involve path planning, needle pose adjusting, needle advancing, etc.^10–12^ However, the corresponding technologies for these tasks are still immature and need to be further progressed, such as spatial registration, positioning and navigation, real-time feedback, respiratory gating, and motion control. In this context, this paper systematically summarized the evolution of commercial PISR systems with a focus on the thoracoabdominal region. Then, the aforementioned technology development are discussed, including spatial registration, positioning navigation, and puncture guidance feedback technology.

The organization of this paper is as follows: Sec. I introduces the research background. Sec. II gives the classification of the representative PISR systems according to the level of automation. Then, the research progress of spatial registration and positioning navigation, puncture guidance feedback, respiratory motion compensation, and motion control are analyzed in Secs. III and IV, followed by the future direction of PISR systems in Sec. V.

## II. PISR SYSTEM REPRESENTATIVES

At present, a series of PISR systems have appeared in clinical applications,^13^ which are introduced sequentially. Considering the coherence and clarity of this paper, the level of automation in positioning and needle insertion is adopted as the classification criterion for different parts in this section. Specifically, the level of automation in positioning and needle insertion is roughly divided into three categories in this paper: manual positioning and manual needle insertion; automatic positioning and manual needle insertion; and automatic positioning and teleoperation for needle insertion. It is noteworthy that, in this paper, tracking refers to the process of moving the end-tip guiding sleeve according to the planned path and needle placement refers to the process of advancing the needle.

### A. Manual positioning and manual needle insertion systems

The robot mentioned in this subsection could plan the puncture needle path, while the path tracking and needle advancing both are manually controlled by the clinicians, as shown in Table I. In this paper, the puncture accuracy is defined as the maximum among the stereotactic accuracy, puncture navigation accuracy, and lesion targeting accuracy. This kind of robot registers the current setup mainly based on the preoperative image information as well as the position information of optical or electromagnetic sensing, before the clinicians manually locating the end-tip guiding sleeve to the entry point assisted by 3D navigation software. Recently, two distinct skeleton graph-based non-rigid registration approaches were proposed using sparse key points^14^ and dense graphs,^15^ respectively, to precisely map preplanned trajectories in intercostal space from preoperative to the current setup.

**TABLE I.** Representative products for PISR.

Year	System	RD institutions	Country	Imaging equipment	DOF	Level of automation	Clinical cases
2014	CAS-One IR	CAScination AG.	Switzerland (CH)	CT/MRI	6	Manual Tracking and Manual Needle Placement	Biopsy
							Ablation
2017	IQQA-Guide MII	EDDA Tech Inc.	America(US)	CT/MRI	5		Biopsy
							Ablation
2021	Savior-L	Accumed Tech Ltd.	China (CN)	CT	5		Biopsy
							Ablation
2021	SGNNO	Hanglok-Tech Co. Ltd.	China (CN)	CT	6		Biopsy
							Ablation
2019	Needle Placement System (NPS)	DEMCON Adcanced Mcchatronics	Netherland (NL)	CT	2	Automatic Tracking and Manual Needle Placement	Biopsy
							Ablation
2016	Micromate	Interventional Systems	Austria (AT)	CT	4		Biopsy
							Ablation
2013	ROBIO EX	Perfint healthcare	India (IN)	CT	5		Biopsy
							Ablation
2014	MAXIO	Perfint healthcare	India (IN)	CT	5		Biopsy
							Ablation
2020	TH-S1	True Health (Guangdong Hengqin) Medical Technology Co. Ltd.	China (CN)	CT	6		Biopsy
							Ablation
2021	Epione	Quantum Surgical	France (FR)	CT	6		Biopsy
							Ablation
2022	SGNIO	Hanglok-Tech Co. Ltd.	China (CN)	CT	6		Biopsy
							Ablation
2021	XACT ACE^TM^	XACT Robotics	Israel (IL)	CT	5	Automatic Tracking and Automatic Needle Placement	Biopsy
							Ablation
2022	VINCENT	Hanglok-Tech Co. Ltd.	China (CN)	CT	6		Seeds implantation

**FIG. 2.**
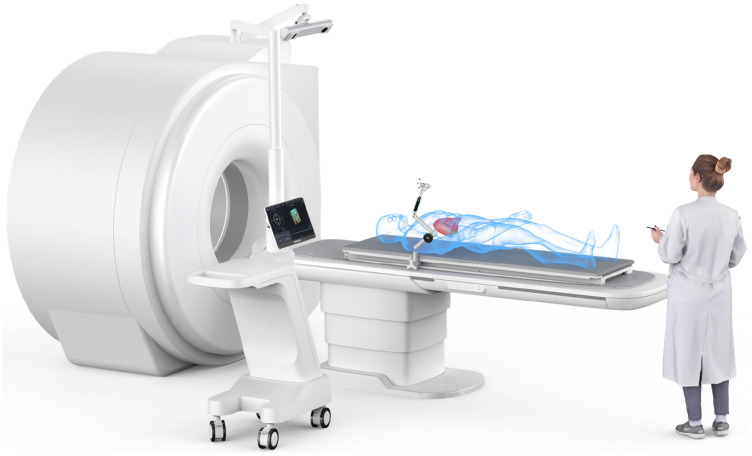
SGNNO, reprinted with permission from https://www.hanglok-tech.cn/ (last accessed January, 2024). Copyright 2024 Hanglok-Tech Co. Ltd.

In the navigation framework combined with optical sensing, the CAS-One IR abdominal puncture surgery navigation system of CAScination AG, Switzerland, is as a commercial representative. With the assistance of CAS-One IR, the clinicians manually match the end-tip guiding sleeve to the entry point of the needle based on the planned trajectory. During the operation, the clinicians manually hold the needle, the accuracy of which is impacted by the issues of hand tremor and fatigue. In addition, due to the lack of real-time positioning information of the end of the needle in the human body, the system cannot accurately get the precise position intraoperatively. As a solution to this problem, the navigation framework with electromagnetic sensing could be considered, enabling the end of the needle to position by sensing devices in real time. However, the physical device makes it inconvenient to be integrated with the multi-degree of freedom manipulators and is extremely susceptible to the influence of magnetic fields. This causes that the corresponding systems are unable to accurately locate the end of the needle *in vivo*, such as IQQA-Guide MII, Savior-L, and SGNNO^16^ (as shown in Fig. 2), created by EDDA Technology Inc., ACCU Med Inc., Hanglok-Tech Co. Ltd., respectively.

For this kind of robot systems, they are not highly automated and rely heavily on the previous experience of clinicians. At the same time, for the operation that lasts for several hours, the surgeons need to bear physical fatigue since an operation always lasts for several hours, having negative effect on surgery to certain extent.

### B. Automatic positioning and manual needle insertion systems

The discussion of robot systems in this subsection could plan a puncture needle path, automatically align the insertion site according to the planned path, and manually advance the needle by a clinician, as shown in Table I. The following content is discussed according to the degrees of freedom (DOF) of the manipulator incorporating non-6-DOF and 6-DOF.

#### 1. Systems with non-6-DOF manipulator

This part mainly introduces the non-6-DOF PISR systems, especially for the robot systems with lower than 6-DOF, including Needle Placement System (NPS), Micromate, ROBIO EX and MAXIO from DEMCON Advanced Mechatronics, Interventional Systems, and Perfint Healthcare, respectively.

Both NPS and Micromate are CT-guided navigation systems for needle positioning. Specifically, NPS is composed of a 2-DOF orientation module and a mechanical device, where the orientation module consists of two stacked rotatable segments and is connected to the CT table by a passive locking module. The insertion angle and depth are planned according to the target and entry point, which is completed manually by the operator. Micromate is a needle positioning and navigation system with 4-DOF, which is previously known as iSYS1^17,18^ and B-Rob.^19,20^ The orientation module of iSYS includes X–Y axis positioning module and needle angle determination module. Based on these modules, the robot moves to the appropriate position with the proper angle according to the planned path. In the same way, the insertion of the needle is also finished manually by the operator.

ROBIO EX and MAXIO are both CT-guided ground-fixed stereotactic puncture positioning navigation systems. Namely, the appropriate needle entry point is recommended by calculating the spatial relationship between the systems of operating table and robot. The relative position of these two coordinate systems is kept unchanged. ROBIO EX is a 4-DOF surgical positioning assistance system, which plans the trajectory of the needle based on the axial relationship between CT images and entry point. Then, the clinician inserts the needle to the expected depth through the needle holder. MAXIO is an upgraded version of ROBIO EX, which introduces a 5-DOF manipulator. However, the insertion of needle is still completed by the clinician. Intraoperatively, multiple CT/MR scans are required to confirm whether the needle has reached the target. This method is with the attributes of long operation time and inability to deal with lesion movement, bleeding, pneumothorax, etc., as well as the huge exposure of radiation for patients.

The non-6-DOF PISR systems with automatic tracking function and manual placement of needle free the operators to some degree and greatly improve the accuracy of the needle positioning. However, the limitation of the puncturing scope makes it difficult to complete complex surgery and the probability of puncturing through the crucial tissues, like lung and stomach, will rise up, increasing surgery risk. Meanwhile, the respiratory motion of the patient would affect the actual position of the soft tissues, resulting in the tissue shifting and deformation as well as the loss of surgical accuracy.

#### 2. Systems with 6-DOF manipulator

This part mainly introduces the 6-DOF automatic positioning robot systems, which assist the surgeons to automatically locate to the entry point of the complex body surface without the need to learn the complicated operation methods of the robot system.

TH-S1 of True Health (Guangdong hengqin) Medical Technology Co. Ltd. includes the following modules: intelligent image analysis and puncture planning, optical navigation, 6-DOF manipulator, and respiratory tracking. The respiratory tracking module collects respiratory information through surface marker signals to finish accurate punctures. Epione developed by Quantum Surgical is a CT-guided 6-DOF PISR system for liver ablation. With the assistance of this system, operators first need to manually move the manipulator to a position close to the surface of the human body. Subsequently, the manipulator can autonomously move to the entry point, following the planned puncture path. Then, the operator manually inserts the needle into the end-tip puncture needle sleeve according to the indicated depth.^21,22^ Epione follows the architecture of ROSA One robots series developed by ZIMMER BIOMET. This series of 6-DOF robots carry out surgical planning through 3D images, tracking the position of the manipulator and surgical instruments in real time.^23,24^ Specifically, the series of ROSA One robot includes the ROSA One Brain neurosurgical surgical robot, ROSA One Spine, and ROSA Keen orthopedic surgical robots.^17,18,23,25,26^ These robots integrate the Polaris VEGA optical sensing module from NDI^27^ and the 6-DOF collaborative robot from KUKA LBR Med (KUKA, Augsburg, DE).^28^ Notably, the manual control could directly offer the tactile cues and force feedback to operators. The robotic assisted radio frequency ablation was compared against the standard manual approach and the study showed that the robotic assisted treatment of liver tumors provided an improvement in terms of the procedure time, procedure accuracy, physician radiation exposure, and patient radiation exposure.^28^ Both the autonomous and the hands-on (i.e., cooperative) control modes for the robotic system were considered to assess the targeting accuracy and no significant difference was observed between the considered control modes.^29^

**FIG. 3.**
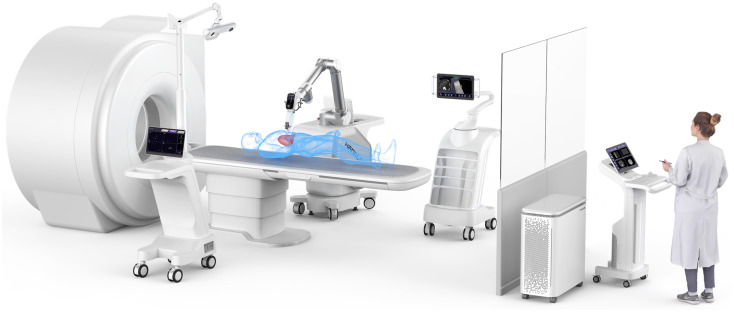
SGNIO, reprinted with permission from https://www.hanglok-tech.cn/ (last accessed January, 2024). Copyright 2024 Hanglok-Tech Co. Ltd.

The 6-DOF PISR has greatly boosted the mobility of the needle and the level of automation of the robots. However, it cannot achieve all the optimal clinical insertion angles. Additionally, several scans are required to verify the needle position, increasing the amount of exposed radiation.^30,31^ The loss of accuracy caused by respiratory movement is still not well solved, either. In the light of these issues to be optimized, the SGNIO shown in Fig. 3 was created by Hanglok-Tech Co. Ltd. with the consideration of the optical positioning, real-time ultrasound imaging, and patients' respiratory behavior.

### C. Systems with automatic positioning and teleoperation for needle insertion

The robotic systems mentioned in this part with the features of planning the puncture needle path, automatically aligning the entry point and complete the advancement of the puncture needle by teleoperation, as shown in Table I. Compared to the robots where clinicians manually advance the onsite puncture needle, teleoperated needle-placement robots have received extensive attention due to their more flexible operation.

XACT Robotics launches a 5-DOF puncture needle navigation system named XACT ACE^TM^ Robotic system. This system is fixed to the patient's body with CT guidance to confirm the entry point and puncture path. The forward and stop of the needle are controlled by a foot pedal to process the insertion of needle. If the needle deviated from the planned path, the robot would correct and adjust the needle according to the current CT image information without repositioning the patient or reinserting the needle. This system also incorporates with a respiratory sensor to compensate for the deviation of puncture accuracy.^32^

**FIG. 4.**
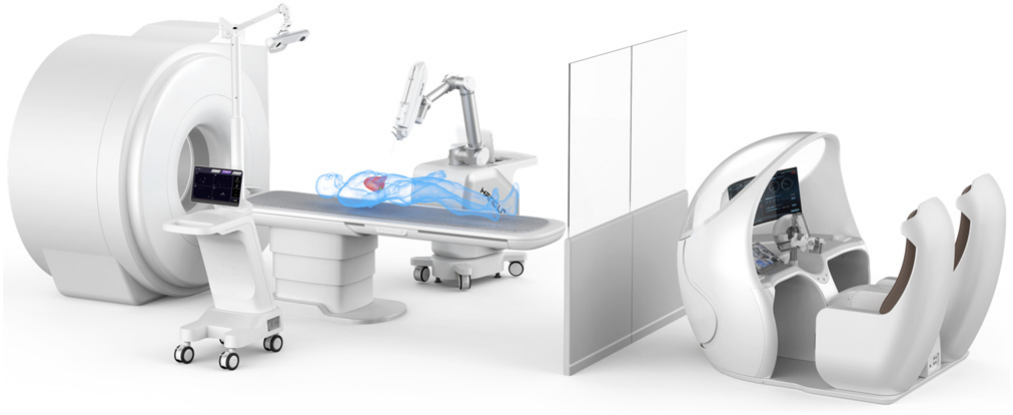
VINCENT, reprinted with permission from https://www.hanglok-tech.cn/ (last accessed January, 2024). Copyright 2024 Hanglok-Tech Co. Ltd.

The robot systems with remote needle placement are first able to reduce the radiation exposure. Furthermore, they also alleviate the errors caused by manual needle placement, such as the breathing, cough, and natural shaking of the operators. Hanglok-Tech Co. Ltd. is now working on a teleoperated multi-functional robot system for seeds implantation—VINCENT, shown in Fig. 4, which fuses the information force, position, and image to provide better sense of immersion. The VINCENT is a master–slave teleoperated multi-functional particle implantation robot system based on a force-position-image hybrid navigation system.

The above-mentioned three types of PISR systems have their own advantages and their level of automation has been gradually raised. However, there are many remained challenges demanding further investigations to enhance the puncture accuracy. Especially when it comes to the patient's respiratory movement, the positioning of needle's end tip, the deformation of the needle, and the uneven force of the needle in the soft tissues, the puncture accuracy cannot always be guaranteed. Actually, none of the current robotic systems can solve these issues well. Therefore, for promoting the subsequent evolution of PISR systems, this paper analyzes and discusses the research status from the view of involved technology, such as spatial registration and positioning navigation, puncture guidance feedback technology, respiratory motion compensation technology, and motion control technology.

## III. SPATIAL REGISTRATION AND POSITIONING NAVIGATION

In traditional interventional surgeries, clinicians should determine the shape, size, location, and surrounding tissues of the lesion by observing the medical images to decide the best entry point of needle and puncture path. This manual operation process requires rich experience of clinicians. Meanwhile, the effect of the puncture surgery mainly depends on the fitting between the actual puncture path and the planned path, since there are several factors that affect surgery precision and treatment results including the positioning of the needle tip, the alignment of needle pose, the instability of manual operations, etc.^33^ Compared to manual operation, robotic-assisted puncture surgery has absolute advantages in ensuring the accuracy and stability. It could assist clinicians in performing surgical operations and compensate the deficiencies of manual operations. The general process of robotic-assisted puncture interventional surgery is as follows: In the preoperative step, segmenting the target and tissues based on medical images, such as lung, stomach, and brain. Then, the puncture path can be automatically planned. Intraoperatively, tissues are rigidly or non-rigidly registered for accurately positioning the target. The surgical plan is accordingly adjusted to guide the robot to reach the planned entry point.

### A. Spatial registration technology

In order to plan paths in the surgery, it is necessary to obtain anatomical imaging information of the human body through images. The 3D visualization of medical images refers to reconstructing 3D models by segmenting and processing preoperative medical image data, like MRI or CT, to obtain digital anatomical models of the patient's lesions. This facilitates surgeons to determine the location of lesions and provides information support for subsequent surgical planning and intraoperative guidance.^34^ Based on the reconstruction of 3D models, a robot-assisted path is planned by integrating preoperative and intraoperative multimodal imaging information, interventional surgical operation requirements, and anatomical features. To derive the optimal path, a two-step strategy was proposed to optimize the most critical settings of minimally invasive interventional surgeries.^35^ An optimization-based approach was developed by formulating constant curvature needle path planning as a nonlinear problem.^36^

Furthermore, the preoperative medical images should be registered to the patient's intraoperative coordinate system. As mentioned before, medical image registration generally includes rigid and non-rigid registration. The methods of rigid registration aim to the rigid organ, which are not influenced by the respiratory motion and widely used in neurosurgery.^37,38^ However, flexible tissues (e.g., chest and abdomen) always suffer motion or deformation because of respiratory behavior. For this issue, the common method is to assume that flexible tissues are rigid and the preoperative positions and gestures of these tissues could be maintained. Inevitably, this would lead to inexact registration results and cannot meet the accuracy requirements. Therefore, the deformation of flexible tissues should be considered and then register to preoperative images through non-rigid medical image registration methods. These methods could be classified into two categories: methods based on physical model and methods based on spatial transformation. Specifically, methods based on physical models treat the image as a physical model and image differences are shown through the physical deformation of model.^39,40^ This kind of method could maintain stable registration effects under ideal conditions, while the physical model is usually a simplification of the real physical world. Namely, the performance of these methods would be degraded when the ideal assumptions are removed and the applicability of these methods is limited. Methods based on spatial transformation are a parameterized method originating from interpolation and approximation theory.^41^ Basic functions are used to represent image deformations as well as combining interpolation algorithms to determine the mapping relationship between images.^42,43^

Deep learning is often adopted in non-rigid medical image registration since it has the ability to perform nonlinear fitting and could achieve higher registration accuracy.^44^ A learning-based image registration framework was proposed to discover compact and highly discriminative features.^45^ In many clinical applications, the fusion of multi-modal images is complex, and a deep similarity learning method was given that trained a binary classifier to learn the correspondence of two image patches.^46^ Traditional registration methods iteratively optimize the objective function of each pair of images to solve the spatial transformation, which has the problems of long registration time and large amounts of calculation. Due to this problem, a bidirectional diffeomorphic registration network was used to improve the accuracy of registration while ensuring the topology-preserving registration, providing a solution to the scarcity of bidirectional registration in traditional convolutional neural networks and local smoothness in the deformation field.^47^ The aforementioned registration methods based on deep learning are still in the experimental stage as they are established on a small amount of sample data and the scope of application is not clear. Hence, it remains to be further explored for registration methods to have both high registration accuracy and reliability.

### B. Positioning navigation technology

The navigation module is the core of a PISR system, which connects the physical and virtual objects in the surgery. With the help of the navigation system, clinicians could have surgical operations, including intuitively estimating the location of lesions, setting entry point, locating target area, and guiding the manipulator to move.^48,49^ The accuracy of positioning the target area plays a decisive role for the effect of surgery. According to the positioning principle, it can be divided into mechanical positioning, electromagnetic positioning, and optical positioning. Among them, the most widely used are electromagnetic positioning and optical positioning, and the latter has highest precision.^50,51^

To be more specific, electromagnetic positioning devices have the advantages of small size, easy measurement, convenient operation, strong penetrability, and high accuracy. A noninvasive magnetic localization method of a helical robot was implemented with a diameter of 1 mm for clearing superficial blood clots, where the localization accuracy is characterized using visual feedback with a position tracking error of 2.35 ± 0.4 mm.^52^ However, the way of electromagnetic positioning is easily affected by metals and magnets. External electromagnetic waves generated by anesthesia machines, high-frequency electric knives, and other equipment will interfere with the positioning effect. In contrast, optical positioning has wider applications due to its ease of use and no electromagnetic interference.^53,54^ For example, based on the CAS-One IR navigation system, Medtronic added a custom needle holding module to locate the needle tip in real time and developed two surgical robot systems, Mazor X^55,56^ and StealthStationTM S8.^57,58^ Mazor X introduced an optical navigation system and a 6-DOF serial manipulator fixed on the operating table. It is compatible with preoperative CT and intraoperative 3D scanning planning modes. Also, it processes 2D perspective projections from the standard C-arm and interacts with 3D imaging to improve accuracy. In order to quantify the accuracy, the actual distance between each coordinate in the space and the vital structure could be introduced, to obtain the minimum distance between each potential path and the vital one.^59^ In addition, StealthStation S8 introduced a 3D camera for tracking instruments and electromagnetic sensors with the ability to fuse different imaging data sources. The function of 360° augmented reality (AR) is added to render the real-time navigation images, allowing anatomy to be viewed from different angles and synchronously enhancing surgical immersion. Likewise, in the optical navigation positioning process, Remebot integrates visual positioning algorithms to automatically complete registration by identifying and locating markers using three cameras and completes surgery manually.^60^ In particular, optical navigation systems are mainly provided by NDI in Canada except for self-development.^61^

However, due to achieve “hand–eye coordination” operation in the robot systems ( “hand” refers to the manipulator and “eye” refers to the navigation system), hand–eye calibration is an essential task.^62^ Hand–eye calibration of robot refers to achieving mapping from optical imaging space to manipulator space by calculating the precise coordinate relationship between “hand” and “eye,” thereby controlling the end effector of manipulator to reach the target position.^63^ The distance error was measured between the real tip of the external probe and the calculated tip to estimate the orientation error.^64^ The angle error between the regressed motion and the real motion was used to evaluate the accuracy of motion direction estimation.^65^ To achieve a relatively ideal accuracy in tracking and positioning, combining MR and ultrasound is a useful approach for medical registration.^66^ Nevertheless, ultrasound guidance limits the accessible area and cannot be used for lung-directed cases because the lungs are full of air. Then, some studies were carried out based on CT. For example, a robotic needle insertion system was proposed using CT images and visual servoing, where the skin entry point and the target to be reached were defined in the CT image and the needle target was then expressed in the robot frame thanks to a 3D registration.^67^ A system composed of CT, a stereo camera, and a robotic system was designed to percutaneously position a needle, where the needle were detected in the triangular region of interest using the Hough transform to identify needle borders.^68^ For providing a high accuracy of the needle position and orientation in real-time to surgeons rather than relying on their individual experience, a method was proposed through making full use of edge points from two sides of the needle in image and creating center points through which an accurate axis was extracted.^69^

## IV. PUNCTURE GUIDANCE FEEDBACK TECHNOLOGY

In PISR systems, the patient may be harmed if the robot control is accurate. In the process of traditional puncture, medical staff often perceives the position of the needle tip by their previous experience. In order for the robot to complete the puncture process automatically, there should be a perception feedback module, i.e., a real-time information feedback module. This module is generally responsible for collecting data from imaging equipment and sensors, and this collected information is then feedback to the controller to further guide the puncture needle.^54,70^

### A. Puncture guidance based on visual feedback

PISR systems are mostly based on visual feedback, utilizing ultrasound devices or camera equipment for navigation guidance during the puncture process. Depending on the imaging method, ultrasound devices provide feedback based on ultrasound imaging, while most camera devices offer feedback using near-infrared optics.

The merits of ultrasound guidance are that it can obtain real-time information with low cost and no radiation. While the drawbacks are the narrow imaging range and the potential needle deviation from the field of view.^71,72^ Amit and ClearGuide both fuse ultrasound images to take advantage of ultrasound images' mobility, providing clinicians with more intuitive image references.^73–75^ Accordingly, the advantages of near-infrared image feedback are its wide imaging range and the entire motion process within the camera's field of view. The disadvantages are the difficulty in obtaining accurate depth information, which can easily lead to the puncture failure.^76,77^ Taking the aforementioned systems as an example, Epione, Mazor X, and StealthStation^TM^ S8 all enhance the positioning and navigation capabilities of the puncture system through the principles of near-infrared light feedback.

To improve the quality of the acquired ultrasound images, the ultrasound confidence map was adopted in an ultrasound-based visual servoing framework.^78^ As an extension of this work, a framework was designed that embedded the confidence-driven control and the ultrasound probe based on the image quality signal.^79^ Also, in order to the high-quality sound propagation within the tissue, a method was presented that aligned the central axis of the US probe to the tissue's surface normal at the point of contact.^80^ To achieve real-time robotic insertion of bevel-tipped flexible needles, a teleoperation framework was considered that enabled the operator to directly and intuitively control the trajectory of the needle tip via a haptic interface.^81^ However, due to the inherent characteristics of ultrasound imaging, a certain contact force between the probe and human is required to optimize acoustic coupling. For manually operating clinicians, maintaining a constant force during ultrasound scanning is challenging. Meanwhile, varying forces would lead to deformation of ultrasound images. As a result, specialized force controllers are needed to maintain contact force during scanning and such controllers are also crucial for preventing excessive force to ensure the safety of patients.^82,83^ It is worth noting that there is only little work that integrates the ultrasound and near-infrared light, which could provide multi-angle navigation for the insertion process and improves surgical accuracy.

### B. Puncture guidance based on force feedback

To achieve more accurate control of the PISR, researchers have begun to add sensors for real-time puncture feedback force detection and explore a variety of feedback mechanisms to improve the control effect. In traditional puncture surgeries, medical staff needs to judge whether the needle tip is aligned with the corresponding target according to their intuition or experience for adjusting the needle insertion angle and speed correspondingly. At present, most of the existing PISR systems only provide visual feedback without force feedback. Therefore, some work introduced force sensors in the perceptual feedback module to obtain more perceptual information to guide the robot to manipulate the puncture needle more accurately.

As an example, a way was proposed to autonomously localize the epigastric region as the starting position for the automatic ultrasound scan with a six axes force sensor fixed to the end effector of the manipulator.^84^ A robotic catheter manipulating system was developed to reduce the performance error and radiation, as well as a torque sensor was applied to measure the torque information during the operation.^85^ In order to stop the scanning process when the pressure on the skin exceeded a preset threshold, two force sensors were attached to the front side of the probe for force measurement.^86^ As an extension, a robotic system with 6-DoF was developed attaching two force sensors to the left and right sides of the front face of the ultrasound probe.^87^ Similarly, an autonomous robotic system for US-guided biopsy of breast lesions was proposed, in which the applied force was measured by a force sensor located in the flange of the robot.^88^ There are some research with the robot's built-in force and torque sensors, further allowing ultrasound acquisitions with optimal skin force and providing repeatable 3D ultrasound volumetric datasets.^89–92^ Although the reported systems generated a contact force by pushing the ultrasound probe onto the body surface directly with actuators and sensors, it is of concern that an excessive contact force can be accidentally applied through control error or because of the failure of actuators and sensors.^93^

### C. FBG-based puncture guidance

Recently, fiber optic sensors have attracted widespread attention due to their characteristics of resistance to electromagnetic interference, excellent biocompatibility, lower cross-sensitivity to temperature, and miniaturized size. This kind of sensor could overcome most of the limitations of electrical sensitive components and achieve precise measurement of surgical instrument deformation in confined space.^94,95^

In order to provide shape sensing of the continuum manipulator, a nitinol wire with an integrated array of 12 Fiber Bragg Grating (FBG) sensors was introduced into the manipulator.^96^ A triaxial distal force sensor for haptic feedback was developed through a novel high-precision FBG based on freedom and constraint topology.^97^ Phase modulated fiber optic force sensing is another significant approach to measure the force.^98^ To be specific, Fabry–Pérot Interferometry (FPI) based fiber optic sensors are convenient for force measurement during MRI guided biopsy procedures because they are MRI compatible and safe for clinical use due to their dielectric nature, chemical inertness, and nontoxicity.^99^

In response to the issue of puncture needles easily undergoing deformation, an approach was developed to reconstruct the needle shape using the strain data obtained from the FBG sensors and then it was as the feedback information for the steering algorithm.^100^ Similarly, to determine the shape of a needle that is being inserted, a method was considered based on the elastic rod theory and Lie group theoretic approach, together with the curvature measurement data obtained by FBG sensors inside the needle.^101^ A reconstruction technique that provides the pose of fiber was presented by converting the measurement from the Bragg gratings to strain and the deduced curvature.^102^ Then, a temperature insensitive calibration model for calibrating needles with FBG sensors was considered to accurately estimate its deformation and a device was designed for simultaneously calibrating the loading direction and shape.^103^ A three-axial force sensor was proposed to measure the pulling force, the steering force, and the lifting force in a flexible endoscopic surgical robot with FBGs embedded in the sensing structure.^104^ Notably, the higher the degree of polynomial fitting, the greater the accuracy required for the position information. Otherwise, any deviation of position information could lead to significant changes in the reconstruction results. Hence, the aforementioned reconstruction method is not suitable for complex deformation reconstruction.

### D. Puncture technology based on flexible needle

In percutaneous interventional surgeries, the most commonly used instrument is the puncture needle, which is mainly divided into rigid and flexible types. Specifically, rigid needles perform punctures in a straight line, but the linear path of needle insertion is restricted. Due to the rigid characteristics of the needle, it is easy to puncture through important blood vessels or nerves during the process. Needle deformations and the patient's breathing can also cause deviations in needle positioning, leading to an inability to reach the target location, severely affecting the effectiveness of surgical treatment.^105^ In contrast to rigid puncture needles, asymmetric bevel-tipped flexible needles experience bending in the body due to forces like tissue-generated action and friction. This allows them to execute curved movements to reach targets within tissues, avoiding vital organs, nerves, and blood vessels.^106,107^ Flexible needles could achieve curved puncture paths since they have lower needle body stiffness, executing more refined interventional operations and reducing surgical injuries. Inevitably, interventional techniques with flexible needles have become a research hotspot recently. They are categorized into passive and active types according to the driving methods. Compared to rigid needles, asymmetric bevel-tipped flexible needles tend to bend as the generated force, such as friction and pressure. This means flexible needles easily bypass vital organs and reach the target during puncture process. Owing to the lower stiffness, flexible needles can follow curved puncture paths, allowing for more sophisticated interventional operations and reducing surgical injuries. Depending on the driving method, flexible needles are classified as passive and active. However, the interactions between flexible needles and soft tissue are complex and related challenges need to be further investigation, such as friction and torsional control as well as the tip pose estimation.^108,109^

On the one hand, passive flexible needles are curved and their puncture path can be altered by changing the base direction or varying the interaction force between the needle tip and tissues. How to achieve low stiffness suitable for flexible organizations is a technical challenge for passive flexible needles. Flexible needle steering techniques offer improved control over implant placement, but often require complex closed-loop control for accurate implantation.^110^ On the other hand, the bending of active flexible needles requires external actuators, such as rope-driven, piezoelectric ceramic-driven, and shape memory alloy-driven. Introducing active flexible needles can add active degrees of freedom to the movement of needle tip. An active needle was proposed for the development of MRI-guided percutaneous procedures, where the needle used a low-transition-temperature shape memory alloy wire actuator to produce bending in the distal section of the needle.^111^ Also, a 3D path planning method to steer flexible needles along curved paths was proposed on the basis of a rapidly exploring random tree strategy.^112^ In order to improve the accuracy of needle placement, the kinesthetic and vibrotactile haptic feedback were combined and a friction estimation algorithm was designed to extract salient information about the cutting force at the needle tip from a force sensor placed at the needle base.^113^ Furthermore, based on the needle shaft radial cross-sectional centroid coordinates, two methods were proposed to estimate the needle tip pose in real-time, which were based on Rodrigues' rotation formula, and Fourier expansion with three-terms and linear interpolation algorithm, respectively.^114^ To further address the issue of needle tip's positioning, ultrasound technology^115^ should be integrated and deployed to plan and execute interventions accurately because of its better reproducibility of measurements,^116^ such as the motion-aware robotic ultrasound imaging technologies,^117,118^ ultrasound deformation correction approaches,^119^ and dual-robot tracking algorithms.^91^

## V. RESPIRATORY MOTION COMPENSATION TECHNOLOGY

Although a large number of puncture robots and systems have been developed, there are still many problems to be investigated for thoracoabdominal PISR systems. The main reason is that the chest and abdomen undergo nonlinear and non-rigid deformation because of respiration. Also, the registration of medical image space and patient space is generally based on rigid matching, which makes it hard to achieve real-time positioning of the target position *in vivo*. As well, there would be a relative movement between needle and tissues due to the respiration. Consequently, it is vital to research respiratory motion compensation technology in robotic surgery.^120,121^

### A. Respiratory motion model

In percutaneous puncture surgery, the bearing condition of the puncture needle is intricate as the complex structure of human skin tissues. To simulate the clinical puncture procedure conducted by clinicians as closely as possible and to compensate for the rigidity shortcomings of robots, some work focuses on establishing respiratory motion models to explore the interaction forces between puncture needle and tissue in soft tissues.^122,123^ To further clarify, the interaction forces mainly comprise of normal force, frictional force, and cutting force.^124^ Depending on the various stages of the puncturing process, the needle bears different forces. When the needle tip punctures the tissue, it is primarily subjected to the normal force. As it pierces through the tissue nearing the target, the main bearing forces are the cutting force from the needle tip passing through the tissue and the frictional force along the direction of the needle shaft. During the needle withdrawal phase, it is primarily subjected to frictional force.^125^

According to the puncture forces during different stages, some work designs patients' respiratory motion models. Instantly, the correlation of the external signal to the motion of the liver in a porcine study using *ϵ*-support vector regression was carried out.^126^ Some work correlates respiratory signal data with internal tumor movement data aiming to predict the real-time position of tumors on the basis of deep learning.^48,127,128^ Yet, these methods were proposed with the assumption that there was a consistent correlation between respiratory signals and the movement of tumors, which might not always be valid from the view of respiratory motion's physiology.^129,130^ On the basis of recent lung motion measurements and the physiologic functioning of the lungs, the motion of lung and lung tumor tissues was modeled with the position of the tissues at a user-specified reference breathing phase, tidal volume, and its temporal derivative airflow (tidal volume phase space).^131^ A robotic needle biopsy technique was introduced, adapting to the patient respiratory pattern and using a robot manipulator to drive the needle toward a moving lung nodule. Then, needle placement was planned to follow an optimal timing and path and was triggered based on the respiratory phase tracking.^132^ To insert accurately needle without stopping the breath, the intermittent insertion control method was proposed, which contributed to decreasing the discomfort and the amount of radiation exposure. Also, a recursive image registration network under deformable respiratory motion was proposed based on ordinary differential equations, where the network learned to estimate time varying voxel velocities to model the deformation in 4D image data.^133^

### B. Target tracking with real-time image

In addition to establishing respiratory motion models, some work proposed strategies to estimate the motion patterns of the target under the influence of respiration in real-time, aiming to reduce the accuracy impact brought by the respiration in the surgery. Currently, multimodality imaging techniques, such as fluoroscopy, ultrasound, and real-time MRI, have been adopted to alleviate this burden. The True Beam system, developed by Varian based on respiratory gating technology,^134^ has been successfully applied in the field of radiation oncology. However, this system is not suitable for thoracoabdominal puncture interventions. Therefore, how to reasonably apply the motion patterns of targets under the influence of respiration to thoracoabdominal PISR systems is still one of the challenges in this field.

A fast CT-CTF deformable registration algorithm was proposed that warped the preprocedural inhale CT onto the intraprocedural CTF for guidance in 3D with incorporating a respiratory motion compensation framework for accurate registration,^135^ where the inhale image, exhale image, and an intermediate phase CT were used for each subject. The shape motion model was designed to enable an unsupervised decomposition of respiration induced high-dimensional body surface displacement fields into a low-dimensional representation encoding thoracic and abdominal breathing.^136^ Furthermore, a real-time matching algorithm to compensate for respiratory motion between a 2D fluoroscopic image and 3D CT images of the lung was proposed, regardless of cardiac motion, based on a newly improved matching measure. This algorithm can improve the accuracy of a guiding system by providing 3D images precisely registered to 2D fluoroscopic images in real-time, without time-consuming respiratory-gated or cardiac-gated CT images.^137^ However, the extraction of real-time image targets and the prediction of moving target areas require high computational costs, which are not conducive to the real-time motion control of robots. Meanwhile, CT imaging takes a long time, making real-time scanning hard to achieve. Also, the diaphragmatic movement caused by breathing has a certain periodicity under CT guidance, while the respiratory cycle is not symmetrical. The exhalation phase within a cycle is generally longer than the inhalation phase, and the movement direction is mainly in the up and down direction rather than the expansion or contraction.

### C. External respiratory signal tracking

Applying markers or sensors on the skin to track the patient's respiratory motion is a common practice because there is a strong correlation between the trajectory of tumor movement and the fluctuation of the chest and abdominal skin surface during breathing. Then, the robot's operation could be adjusted correspondingly.^130,138–141^

The relationship between internal and external marker positions was continuously accounted for and was regularly checked and updated, where a polynomial model was studied to describe the relationship between internal tumor motion and external (chest/abdominal) marker motion.^142^ As a commercial representative, three external markers were used by CyberKnife, developed by Accuray, to capture the patient's respiratory characteristics, establish the correlation function between markers and tumor locations, and then estimate the location of the tumor.^143^ Also, a method was proposed to the 4D active tracking and dynamic delivery incorporating the tumor motion prediction technique, which was applied to the two commercially available robotic treatment couches.^144^ Furthermore, a marker-based depth frame registration technique was introduced to limit the measuring area into an anatomically consistent region, handling the patient's movement during the treatment.^145^ The approach to extract highly representative surface motion features based on selecting moving surface areas was considered. Similarly, the surface area was segmented into several regions before measuring their corresponding correlations with internal tumor motion according to high Pearson's correlation coefficients.^146^ It is noted that the estimation of the tumor position requires extracting the respiratory motion characteristics of the chest and establishing correlation models manually, which is always affected by the placement and number of markers. As a result, point clouds of the chest and abdominal skin surface were employed and a hierarchical network was built to extract the features of the skin and map those features to the location of tumors.^147^

## VI. MOTION CONTROL TECHNOLOGY

Surgical robots have complex dynamics, strong coupling between degrees of freedom, and nonlinear characteristics, such as hysteresis, which need to rely on advanced control technology to achieve their precise operation. At the same time, master–slave control is a common control method of surgical robots, which can not only give full play to the advantages of precise control of the robot but also fully combine the clinical experience of the surgeon. This section focuses on the analysis and discussion of the manipulator motion control and needle motion control involved in the puncture surgery robot.

### A. Manipulator motion control technology

To achieve precise control of the manipulator, innovative algorithms based on classical motion control theory provide a fundamental guarantee for the flexible movement and reliability. For instance, a haptic teleoperation system with force feedback and a bilateral controller method was suggested, where the master control used impedance control and the slave control used a sliding mode control.^148^ For collaborative operations under force-position hybrid control, a robot trajectory planning for edge detection and a collaborative control strategy with constant force were proposed.^149^ As a matter of fact, real robotic systems are complex to model owing to the nonlinear and time-varying property. To deal with this issue, an active-model-enhanced control scheme was developed to enhance a PID controller, and the modeling errors was eliminated by integrating an active modeling algorithm into this controller.^150^ A way of controlling the position of the active arms of a Da Vinci-type surgical robot was considered and a camera was placed on the slave system to provide these positions information, then the presence of medical staff to handle these arms manually is no longer necessary.^151^ Also, a perception and control framework were achieved that used real-time tactile feedback to accomplish the task of following a dangling cable, in which an linear-quadratic regulator controller based on a learned linear model of the cable sliding dynamics was combined.^152^

In the context of master–slave control, a dual-loop control system was considered, including closed-loop inverse kinematics and adaptive control with optimal control parameters, for the slave robot to track a random trajectory upon updating the dynamic parameters of the slave robot and this system could be potentially utilized for actual teleoperated surgery and surgical training.^153^ Then, the lack of maneuverability for physicians when operating master–slave robots is also a significant challenge.^154^ Therefore, a control scheme of master–slave manipulators that provided the ideal kinesthetic coupling such that the operators can maneuver the system as though they were directly manipulating the remote object themselves.^155^ On the basis of requirement analysis of manual operations, a master–slave robotic system mainly consists of a 3-DOF slave manipulator and a 3-DOF master manipulator to achieve intuitive motion mapping and proper ergonomics.^156^ To provide the operators with high-fidelity force feedback, a thorough modeling approach based on successive isolation of system components was investigated.^157^ Similarly, a force feedback hand controller system was shown, including a 3-DOF translational and 3-DOF rotational hand controllers, respectively, to implement position and posture teleoperation of the robot end effector.^158^ Considering the stability issues, a type of the force reflection algorithms was studied on the stability and performance of a dual-arm haptic-enabled teleoperator system both with negligible as well as non-negligible communication delays.^159^ A forward and inverse kinematics model of the slave hand and a master–slave motion control algorithm were designed based on the screw theory and motion model to boost the hand–eye coordination consistency of the surgical robot system, by integrating consistency motion control, relative position control and proportional control.^160^

Master–slave robots have certain advantages in improving surgical accuracy, while the careful attention is required to avoid dangers from inappropriate operations.^161^ To have fast tracking performance and strong robustness, a variable universe fuzzy PID method was adopted to improve fuzzy PID method's control precision without reducing the speed of response.^162^ Furthermore, for enhancing the precision and comfort of operations, a physical human-robot interaction control scheme of the haptic master manipulator was used with a torque observer based on generalized momentum.^163^ To learn the finger motions and to determine when to lift an object, an algorithm was trained by combing fingertip tactile sensing, joint torques, and proprioception as well as a multimodal agent through reinforcement learning.^164^ As for the aforementioned haptic feedback, it has been investigated by many researchers. This technology could not only help surgeons minimize potential side effects but also reduce errors in remote operations, tissue damage, and operation time.^165–167^

### B. Puncture needle motion control technology

During the whole puncture operation, the key issue is how to control the puncture needle to reach the target point while avoiding important tissues, so the motion control of the puncture needle is particularly important. Based on the basic concepts of puncture needles (see Sec. IV D), this subsection first discusses the motion control of flexible puncture needles. There are two main modes of motion control for flexible puncture needles: teleoperation control and autonomous control.^168^

Teleoperation control mainly involves manipulating the robot's master arm to control the slave arm located at a distant end, thereby accomplishing the advancement and rotation of the needle. Traditional control algorithms have the advantages of simplicity, robustness and ease of control and a PID algorithm was proposed to control the needle's motion.^169^ While these traditional methods cannot satisfactorily meet the puncture performance since the needle is always subjected to the force of human tissue. In the presence of tissue deformation and uncertainty, a feedback controller was designed to steer a needle along 3D helical paths and vary the helix radius to correct for perturbations.^170^ Then, an approach keeping the physician in the loop to control insertion speed was proposed, where the tip was automatically guided to a planar slice of the tissue during the insertion process.^171^ To maintain the needle tip in a desired plane in 2D control, three estimation algorithms for estimating the states of the needle tip were employed to measure the position and the posture of the needle in the process of puncture.^172^

Autonomous robots primarily rely on real-time imaging to independently perform the puncture surgery. Since this scenario relates to safety and ethics, there are many issues to be resolved before it can be practically implemented. The early puncture robots are mainly single DOF, which could only be used as an experimental platform. For achieving higher precision and smoother actions as well as completing more complex tasks, robots should have more DOF, for example, 3-DOF puncture mechanisms,^173^ 4-DOF manipulators operating needle to complete insertion actions,^174^ 5-DOF,^175,176^ and 7-DOF robots.^177^ To accurately guide the needle toward inner body targets, a model-based nonlinear observer was adopted for partially estimating the needle tip orientation by image-based position measurements.^178^ However, this method can only obtain the pitch angle and rotation angle of the needle, and the absence of yaw angle is a limitation. Benefit of flexible echogenic needles for robotized guidance under 3D ultrasound was quantified for improving needle visibility and detection robustness, where echogenic term refers to the etching of microstructures on the needle shaft.^179^ Similarly, a control law using task functions was proposed to reduce targeting error and forces applied to the tissue, steering via base manipulation and tip-based steering.^180^ Then, a path planning and tracking control method for flexible needle steering in dynamic environment was proposed based on the artificial potential field algorithm and curve fitting algorithm.^181^

## VII. DISCUSSION AND CONCLUSION

Robotic systems are renowned for their precision in spatial positioning, high stability, dexterous maneuverability, and operational endurance. The integration of robotics with medical technology holds significant clinical value and represents a current direction of development in the field. In this review, we highlight commercialized PISR systems that are at the cutting edge of technology and advancements. Despite the remarkable capabilities of these robotic systems, they are not without their limitations and challenges, particularly in addressing the complexities of soft tissue deformation and the intricacies of patient respiratory movements. To pave the way for the next-generation of PISR systems, a concerted effort toward evolutionary advancements is necessary and several key directions could be considered: 1. Enhanced multi-dimensional perception: future PISR systems should harness information across a spectrum of dimensions, not limited to but including optical information, force feedback, positional insight, and visual cues. An integration approach to holistic information is essential for achieving a comprehensive understanding of the surgical environment. 2. Advanced information acquisition: the incorporation of ultrasound technology into navigational systems, when synergized with AI or advanced LLM techniques, promises a leap in the robots' perceptual capabilities. This combination is poised to unlock a new level of precision and awareness within robotic systems. 3. Hardware innovation: addressing the need for minimizing patient trauma, especially in procedures requiring multiple puncture needles, calls for the innovation of robot hardware structures, to broaden the applicability of robots in complex puncture procedures.

## Data Availability

Data sharing is not applicable to this article as no new data were created or analyzed in this study.
